# Supplementary material for: A randomized controlled trial on anonymizing reviewers to each other in peer review discussions
Source: PLoS One. 2024 Dec 27;19(12):e0315674. doi: 10.1371/journal.pone.0315674 (PMC11676492; doi:10.1371/journal.pone.0315674)
Supplement: S1 Appendix — (PDF) [file pone.0315674.s001.pdf]

Appendices

A Assessing politeness of discussion posts

To assign a politeness score to each text, without compromising the privacy of the peer review data, we used a local implementation of a large language model. Specifically, we implemented the most recent (as of August 2023) and quantized version of the largest variant of Vicuna, `vicuna-13B-v1.5-GPTQ`, with a context length of 4,096 tokens. We set the temperature to be 0.7, and limit the generation output to numbers $\{1, 2, 3, 4, 5\}$.

To run the Vicuna model, we first downloaded its model weights using the `TheBloke/vicuna-13B-v1.5-GPTQ` model checkpoint from huggingface. To run the model in inference mode in order to generate the politeness scores, we used the Python package `ExLlama`.

We carefully craft a few-shot learning based prompt with three examples chosen from the politeness dataset provided in [33]. Our overall prompt design consists of the elements described in Table 4. Each prompt concatenates all the elements in order, namely Instruction + Examples + Query. The discussion text to be assessed is added in place of `[post]` to complete the prompt. Further, we limit the generation output to integers 1–5, by disallowing tokens other than 1–5 using the function "`ExLlamaGenerator.disallow_tokens()`."

Element Type	Text
Instruction	We are scoring reviews based on their politeness on a scale of 1-5, where 1 is highly impolite and 5 is highly polite.
Example 1	Review: Please say in the main text that details in terms of architecture and so on are given in the appendix.<br>Politeness score: 5
Example 2	Review: From this perspective, the presented comparison seems quite inadequate.<br>Politeness score: 3
Example 3	Review: Please elaborate on how you end up in this mess.<br>Politeness score: 1
Query	Review: `[post]`<br>Politeness score:

Table 4: Our prompt to query the politeness score of reviewers' posts consists of the following: (1) an overall instruction to describe this politeness scoring task, (2) three examples for LLM to understand this task better, and (3) query of the politeness score given a post by a reviewer.

To be robust to the biases shown by generative models in their output, due to the ordering of the few-shot examples, we do the following. We create six paraphrases for each post by alternating the order of the three examples used in the prompt. That is, we concatenate the three examples in six different ways: (1) Examples 1 + 2 + 3, (2) Examples 1 + 3 + 2, (3) Examples 2 + 1 + 3, (4) Examples 2 + 3 + 1, (5) Examples 3 + 1 + 2, and (6) Examples 3 + 2 + 1. As we take mean over the outcomes of each of these

paraphrases, we ensure that the final generated outcome is not biased due to the ordering of the examples.
