# Supplementary material for: A randomized controlled trial on anonymizing reviewers to each other in peer review discussions
Source: PLoS One. 2024 Dec 27;19(12):e0315674. doi: 10.1371/journal.pone.0315674 (PMC11676492; doi:10.1371/journal.pone.0315674)
Supplement: S2 Appendix — (PDF) [file pone.0315674.s002.pdf]

B Mann-Whitney test details

B.1 Mann-Whitney U test for survey responses

To test for difference in the self-reported experiences of reviewers discussed in Section 4.4, we employ the Mann-Whitney U test. For each survey question, given the responses collected, we want to test the hypothesis described next. Let X^a indicate the random variable sampled as a response from a reviewer in the non-anonymous condition, and $X^{\widetilde{a}}$ indicate the random variable sampled as a response from a reviewer in the anonymous condition. We define ordinal comparisons between the responses as

Strongly agree > Somewhat agree > Neither agree nor disagree > Somewhat disagree > Strongly

Under this ordering, for each survey question, we want to test the hypothesis:

$$\begin{aligned} H_0 &: \mathbb{P}(X^a > X^{\widetilde{a}}) = \mathbb{P}(X^a < X^{\widetilde{a}}) \\ H_1 &: \mathbb{P}(X^a > X^{\widetilde{a}}) \neq \mathbb{P}(X^a < X^{\widetilde{a}}). \end{aligned} \tag{2}$$

Let us denote the response data available for a question in the anonymous and the anon-anonymous condition as: $\{x_1^a, \cdots, x_{n_a}^a\}$ and $\{x_1^{\widetilde{a}}, \cdots, x_{n_{\widetilde{a}}}^{\widetilde{a}}\}$ respectively, where $n_a, n_{\widetilde{a}}$ indicate the number of respondents in the corresponding condition, and x_i^a and $x_i^{\widetilde{a}}$ indicate the ith responses therein. With this notation, we lay out the step-wise procedure for conducting the Mann-Whitney U test for (2).

- Step 1: Compute the Mann-Whitney U-statistic. This statistic measures the frequency of a response in one condition being higher than a response in the other condition with ties counted as half. This is encapsulated in the following function

$$S(a,b) = \begin{cases} 1 \text{ if } a > b, \\ 0.5 \text{ if } a = b, \\ 0 \text{ if } a < b. \end{cases} \tag{3}$$

  Note that this is a non-symmetric function, since $S(a,b) = 1 - S(b,a)$. The Mann-Whitney U statistic is defined symmetrically as

$$U = \min\left\{ \sum_{i=1}^{n_a} \sum_{j=1}^{n_{\widetilde{a}}} S(x_i^a, x_j^{\widetilde{a}}), \sum_{i=1}^{n_a} \sum_{j=1}^{n_{\widetilde{a}}} S(x_j^{\widetilde{a}}, x_i^a) \right\}. \tag{4}$$

  We compute the normalized U-statistic as $\dfrac{U}{n_{\widetilde{a}} n_a}$.

- Step 2: Compute the p-value using a permutation test.

  Let γ indicate the number of iterations. We set $\gamma = 100000$. For each iteration, we randomly shuffle the data across the two groups $\{x_1^a, \cdots, x_{n_a}^a, x_1^{\widetilde{a}}, \cdots, x_{n_{\widetilde{a}}}^{\widetilde{a}}\}$ such that we have n_a and $n_{\widetilde{a}}$ samples respectively in the hidden and the shown group. Then for $k \in \{1, 2, \ldots, \gamma\}$, compute U_k based on shuffled data according to (4). Then, we compute the two-tailed p-value as

$$p = \frac{2}{\gamma} \min\left\{ \sum_{k=1}^{\gamma} \mathbb{I}(U_k > U) \quad , \quad \sum_{k=1}^{\gamma} \mathbb{I}(U_k < U) \right\} \tag{5}$$

B.2 Mann-Whitney U test for politeness scores

To test for difference in politeness of discussion posts across the two conditions, we consider posts written by senior reviewers separately and junior reviewers separately to account for selection bias. Let $P^a \in \{p_1^a, p_2^a, \ldots\}$ denote the set of politeness scores assigned to anonymous discussion posts by senior reviewers, and similarly $P^{\widetilde{a}} \in \{p_1^{\widetilde{a}}, p_2^{\widetilde{a}}, \ldots\}$ denotes scores for non-anonymous discussions. The set of scores assigned to posts by junior reviewers are denoted by $Q^a \in \{q_1^a, q_2^a, \ldots\}$ and $Q^{\widetilde{a}} \in \{q_1^{\widetilde{a}}, q_2^{\widetilde{a}}, \ldots\}$ for the anonymous and non-anonymous condition respectively. To account for difference in behaviour across seniority groups, we define the normalised U-statistic as

$$\begin{aligned} U_{PQ} =& \frac{\left(\sum_{p^a \in P^a} \sum_{p^{\widetilde{a}} \in P^{\widetilde{a}}} \mathbb{I}\left(p^a > p^{\widetilde{a}}\right) + 0.5\mathbb{I}\left(p^a = p^{\widetilde{a}}\right)\right)}{|P^a||P^{\widetilde{a}}| + |Q^a||Q^{\widetilde{a}}|} \\ &+ \frac{\sum_{q^a \in Q^a} \sum_{q^{\widetilde{a}} \in Q^{\widetilde{a}}} \left(\mathbb{I}\left(q^a > q^{\widetilde{a}}\right) + 0.5\mathbb{I}\left(q^a = q^{\widetilde{a}}\right)\right)}{|P^a||P^{\widetilde{a}}| + |Q^a||Q^{\widetilde{a}}|}, \end{aligned} \tag{6}$$

where $\mathbb{I}(\cdot)$ denotes the indicator function. To derive the significance of the test, we conduct a permutation test as described in Step 2 in Section B.1 except when the data is shuffled in each iteration, the elements of P^a are shuffled at random with elements of $P^{\widetilde{a}}$ and the elements of Q^a are shuffled at random with $Q^{\widetilde{a}}$.
