# Supplementary material for: A randomized controlled trial on anonymizing reviewers to each other in peer review discussions
Source: PLoS One. 2024 Dec 27;19(12):e0315674. doi: 10.1371/journal.pone.0315674 (PMC11676492; doi:10.1371/journal.pone.0315674)
Supplement: Attachment — Submitted filename: response.pdf [file pone.0315674.s003.pdf]

Our responses are in blue and the original reviewer comments are in black font.

Journal Requirements:

We have made the revision according to the style of PLOS ONE and reviewed the reference list. We have removed funding information from the acknowledgments. The funding statement on file is correct.

Editor's comments: As per my e-mail of June 17th, the abstract needed to be cut back to the word limit, with numbered and bulleted points removed. I did receive your e-mail explaining that this had been done but that you had not received a reply from the Journal regarding submitting the corrected paper.. I was also trying to get the system reopened to allow for the resub, but eventually in the interest of time I sent the paper out for review with the abstract as submitted. I trust that your paper will be resubmitted with the abstract correctly formatted and within the word limit. In addition to this, please pay careful attention to the points raised by two reviewers below.

Thank you very much for handling our manuscript and your feedback. We have uploaded the shortened abstract in the revision. We have incorporated the comments of the reviewers in the revised manuscript, and provide our responses to each of their comments below.

Reviewer 1:

This is a very comprehensive analysis for a review process that is not very common, at least when assessing scientific articles and conference abstracts: a review process with internal discussions between the reviewers. I only know this from juries that have to decide on prizes that are beyond scientific. Therefore, in my opinion, the relevance of the research question is not very high. It turns out that the differences between anonymous and non-anonymous discussions are generally small. This is quite a surprising and important finding. However, it does not correspond to the general statement made by the authors ("Overall, the experiment reveals evidence supporting the advantages of an anonymous discussion setup in the peer-review process"). It would be more correct to state that differences were either not noticeable at all or were very small in favor of anonymous discussions.

Response: Thank you for your response, we appreciate your feedback on our work. We would like to clarify that conferences in fields related to artificial intelligence, which review many tens of thousands of papers every year, typically do have such discussions. We would also like to clarify that these conferences review full papers (not just abstracts) and are frequently the terminal venues of publications. In addition, discussions between reviewers also takes place in various grant panel reviews (although these occur in face-to-face settings rather than typed forums).

Based on your useful feedback on the abstract, we have edited the statement in abstract to:

"Overall, this experiment finds small and significant differences favoring the anonymous discussion setup based on the evaluation criteria considered in this work."

Reviewer 2:

Firstly - "thank you", this is an interesting topic that ought to interest all researchers / reviewers / editors (effectively a global audience, beyond UAI). That said, for those unfamiliar with the multi-stage decision making that underpins this survey, it might be helpful to have a graphical representation of the study design (a la CONSORT).

What is not clear, is the determination of acceptance - the point at which a paper is included and specifically, how its "score" is computed. Do reviewers trade/haggle/negotiate/interact as a result of intermediate typed comments? How is the endpoint/consensus determined ? The reason for asking this, is that downstream you report the extent to which reviewers converge with final scores and this mechanism does not seem to be included in the descriptive account of the study. Crucially, the reader needs to know how the final score is computed.

Thank you for your feedback and your very kind words of encouragement! We really appreciate all your extensive and all very valuable feedback on our manuscript.

The determination of the final acceptance of the paper is then done by the meta reviewer based on the initial reviews, the authors' responses, these discussions, and potentially the meta reviewer's own perceptions of the paper. We have now clarified this in the revised manuscript.

We agree with you that a visual aid would be a helpful augmentation to the description of the specifics of the review process in Section 3. Based on your suggestion, we have added the following image to the manuscript in Section 3. Thank you very much for this very helpful feedback!

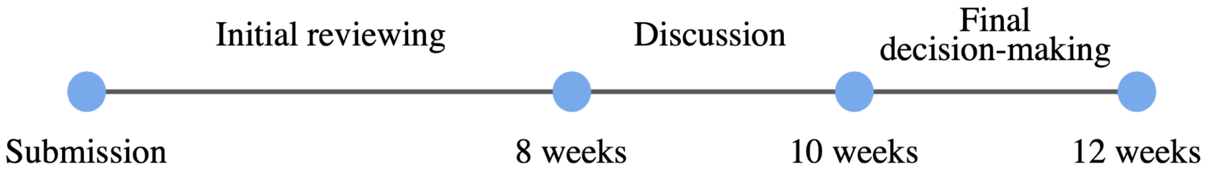

Caption: Timeline of the peer-review process of typical machine learning and artificial intelligence conferences. After the release of initial reviews, authors of papers and the reviewers have an asynchronous discussion. Finally, meta-reviewers aggregate the results of the review process into final decisions. The duration of each stage varies across conferences, and this figure corresponds to the UAI 2022 review process with the duration of each stage rounded to weeks.

Out of interest, did any of the authors take on the role of reviewers - and if so, then what were their NA / A status?

None of the authors of this work participated in the UAI 2022 review process as reviewers.

Although the primary focus of the study relates to the issue of reviewer anonymity, this is intimately connected to that of reviewer seniority and this strand of investigation is not consistently followed in all reported analysis.

The survey we conducted seeking preferences and experience of reviewers was anonymous, hence there is no data available regarding the related seniority status of the responses obtained.

The remaining analyses concern the number of messages posted by reviewers and the politeness of reviewers. In the analysis concerning messages posted by reviewers we explicitly discuss the role of seniority.

Based on your feedback, we now explicitly provide analyses related to each seniority level when considering politeness of reviewers. Specifically, we added mean politeness scores obtained by posts written by different seniority levels to the revised manuscript. We reproduce the corresponding text here for your reference:

"Across different seniority levels, we see that in the anonymous condition, senior and junior reviewers receive a mean politeness score of 3.68 and 3.79 respectively. Meanwhile, in the non-anonymous condition, senior and junior reviewers receive an average politeness score of 3.66 and 3.77 respectively. "

Figure 1 reveals a key issue. The modal number of discussion posts is zero, with a marginally higher proportion in the A group. This type of behavioural data invites us to consider what constitutes the most appropriate measure of "average". Is it meaningful to report an average response of 0.53 given the overwhelming majority of nil posts?
Once we remove the nil response group, it seems that there is parity for category 1, with a trend towards greater number of interactions in the A group vs NA group. Surely this is a 2-step issue, what is the initial response - (post or not post), then consider what is going on amongst those who DO post. It'd be so more informative if we knew whether there is any difference in post/no-post rates by seniority. Is it feasible to redesign Figure 1 to show this? Perhaps with a data table? The text states that a permutation test with 1,000,000 iterations was used to test for significance - this just feels like an overkill, especially given the resulting (marginal) p-value. There are other ways of assessing concordance in ordinal data of this type - might a contingency table be more informative?

Thank you for pointing this out. Following your useful suggestion, we now also consider, for each paper-reviewer pair, a binary indicator variable to indicate whether there was any post or not and test for significant difference in this variable across the two conditions. We further stratify the paper-reviewer pairs with a post based on the seniority of the reviewer. The results are discussed in Section 4.1, and we reproduce the text here:

“Given the low rates of posting overall in both the conditions, as evidenced by the frequency of zero in Figure 2, we consider a binary indicator variable indicating post or no post. Precisely, for each paper-reviewer pair this indicator is set to one if there exist any posts associated with the pair and zero otherwise. We then test for significance of difference across the two conditions using Fisher's exact test. The outcome contingency table is depicted in the last two rows of Table 1 and the corresponding two-tailed p-value is 0.43. This implies no significant difference between the two conditions in posting vs not posting.”

The contingency table is also reproduced here, in the revised manuscript we have added it to Table 1:

	Anonymous	Non-anonymous
Number of {paper-reviewer} pairs with posts	419	385
Number of {paper-reviewer} pairs without posts	744	733

Updated text on stratification of posting behavior based on reviewer seniority:

“Next, we look at the distribution across seniority level within the paper-reviewer pairs where at least one post was made by the reviewer for the paper. Therein, in the non-anonymous condition, senior reviewers made 214 of the total 385 paper-reviewer pairs, which is approximately 56%. Similarly, in the anonymous condition, senior reviewers made 230 of the total 419 paper reviewer pairs, which is approximately 55%. Thus, within paper-reviewer pairs with posts, we see that senior reviewers participated more than junior reviewers, however their participation was similar across the two conditions.”

This issue of "closest" fit to acceptance score .... might it be informative to take each pair and compute the differences for the senior & junior reviewers, code the winner / loser as 1/0 respectively and then run a sign test?

We understand that there are many possible ways to run this analysis. However, executing multiple analyses on the same data towards the same research question will lead to a multiple testing problem, and accounting for multiple testing would dilute the significance of each individual test. With this in mind, we predecided on what we thought was the most suitable test given the data available, in order to avoid multiple testing (and associated power reduction) issues. Thank you very much for the suggestion though, and we hope the reviewer understands our rationale.

I completely LOVE the politeness index section - however, it does introduce a massive confounder in that you/we all have to "believe" in ChatGBT. Personally I think it's novel but risky and wouldn't want to go to the wall in its defence just now. You might want to deal with this in the discussion? To be honest, I could see this being taken out of this current manuscript to form the basis of a 2nd separate paper. Just a thought.

Thank you very much for your very kind remarks on our work! You bring up an important point, we have added it to our discussion section. We reproduce the text below. Additionally, we note that we followed standard practices and carried out checks to ensure the results of ChatGPT were not random or biased–

(1) To mitigate bias due to the ordering of the few-shot examples, we created six paraphrased versions of the prompt by varying the order of the examples.
(2) Since the output generated by LLMs can be different across iterations, each version is queried ten times, and the mean across all paraphrases and iterations is used to indicate the final politeness score.
(3) We validated the consistency of the generated scores by measuring the correlation of scores generated for the same prompt across iterations. This check was run over 1125 unique prompts. We found a significant correlation between two randomly picked iterations for each prompt query, with a Pearson correlation coefficient of 0.42 and a two-sided p-value smaller than 0.001.

We reproduce here the updated text from discussion:
“Next, we discussed in Section 4.3 our implementation of LLM-based politeness analysis. Here, it is important to note that given the variability of performance of different LLMs and subjectivity of politeness evaluation, despite the robustness checks conducted in Section 4.3, the results could vary with a different approach to assigning politeness scores to review texts.”

When categorical response data are "scored" (as in #4.5) and then treated as cardinal, being represented by a MEAN (with 2 decimal places) then I do wonder what is going on. The reader is entitled to see at least, a descriptive account of the raw data. So, in this case it would improve matters if we have a revised form of Table 3 that shows the frequency of response category for each of the 5 statements, separately for N/A and A groups. We can then visualise the distribution for each statement and extent of any similarity between statements. Of course, it'd be simple to reslice the salami and checkout the patterns for senior/junior reviewers.

The "scoring" system imposes the researchers ex post value on responses. Hence the statement "I strongly prefer reviewer identities to be HIDDEN from other reviewers" (value=>2) reported by a single reviewer, entirely negates the statement "
"I weakly prefer reviewer identities to be SHOWN to other reviewers" (value =>-1). Please review this aspect of the analysis and consider alternative ways to handle these data?

I prefer the reporting format in Figures 3 & 4 which deal with a somewhat similar data type.

We have added a visual representation to show what the data comprises. This will aid readers' understanding of the distribution of the data. Thank you for the note. We reproduce the added figure and corresponding text (in Section 4.5) here for your reference:

“Furthermore, we visualize the distribution of the responses in Figure 5, separated based on the group of the reviewers. We see that reviewers in the anonymous condition are skewed in their overall preference in favor of the anonymous condition. Meanwhile, meta-reviewers and reviewers from the non-anonymous condition are relatively more equally distributed across the two overall preferences.”

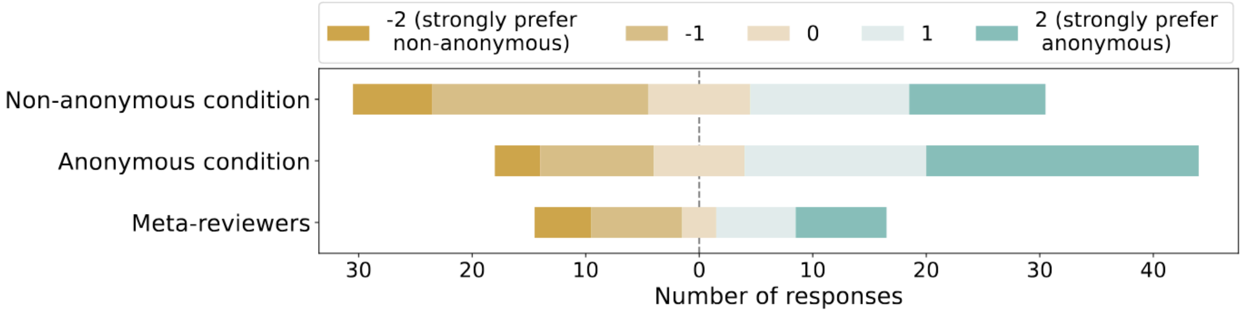

Figure 5.
Caption: Survey outcomes on reviewers' and meta-reviewers' reported overall preference between the two conditions in the experiment. Respondents provided Likert-style responses ranging from ‘I strongly prefer reviewer identities to be shown to other reviewers' to ‘Indifferent' to ‘I strongly prefer reviewer identities to be hidden from other reviewers.'

It might be useful in the Discussion section to consider whether any aspects of the design might have been modified, as well assessing the value of any findings of note, for example in guiding best practice in conference abstract reviews. One suggestion that I would have considered is the use of some well-designed abstracts with specific methodological/data reporting "flaws" to test the extent to which such material is differentially noted; additionally, if UAI conference abstract design so permits, how about infiltrating (hidden) identification of authors by loading the abstract with references to their papers?

Thank you very much for your suggestions. To clarify, the UAI conference did not review just abstracts, but full papers. In the field of computer science, conferences review full papers (not only abstracts), are frequently a terminal venue of publication, and are often rated higher than journals. That said, we agree with you that a way to measure the end-to-end effectiveness of the peer-review process (under any policy or intervention) would be to write down the objectives of the peer review process, construct submissions with objective ground truth, and evaluate reviews as compared with this ground truth. In the revised version, we include a discussion about this at the end of the paper, and this text is reproduced below:

"To fully assess the effectiveness of any such interventions or policies in the peer-review process, an alternative approach would be to establish clear objectives for

the process (e.g., identifying flaws in papers), create submissions with objective ground truth, and evaluate reviews against this benchmark. Along these lines, some past work focuses on identifying flaws in full papers [Baxt et al., 1998, Emerson et al., 2010, Godlee et al., 1998, Schroter et al., 2004, 2008], [Shah, 2022, Section ‘Ensuring Correctness’] and evaluating LLMs’ ability to identify flaws in papers and identifying abstracts with superior results [Liu and Shah, 2023],[Shah, 2022, Section ‘AI Reviewing’]. However, implementing this in real peer-review processes poses several challenges. It requires constructing complete research papers and then requires many reviewers to spend time evaluating these papers. While we strongly advocate for more experimentation to enhance the understanding of the peer-review process and inform evidence-based policy adjustments – especially since substantial researcher time is spent on it – the decision to undertake such efforts ultimately rests with organizers and the research community, who must weigh the additional workload against the potential benefits.”
