# Supplementary material for: A randomized controlled trial on anonymizing reviewers to each other in peer review discussions
Source: PLoS One. 2024 Dec 27;19(12):e0315674. doi: 10.1371/journal.pone.0315674 (PMC11676492; doi:10.1371/journal.pone.0315674)
Supplement: Attachment — Submitted filename: Review response (UAI 2022) (2).pdf [file pone.0315674.s004.pdf]

Thank you very much for your feedback on the manuscript, and your suggestions which we greatly appreciate! Our responses are in blue and the Editor's comments are in black font.

Editor's comments:

I have considered your revised manuscript and I am satisfied that you have effectively taken care of the issues raised by the reviewers.
I would like to suggest two minor changes before moving forward.

1- The first point raised by Reviewer 1 included this statement:

> *This is a very comprehensive analysis for a review process that is not very common, at least when assessing scientific articles and conference abstracts: a review process with internal discussions between the reviewers. I only know this from juries that have to decide on prizes that are beyond scientific. Therefore, in my opinion, the relevance of the research question is not very high.*

…and your response to this was:

> *We would like to clarify that conferences in fields related to artificial intelligence, which review many tens of thousands of papers every year, typically do have such discussions. We would also like to clarify that these conferences review full papers (not just abstracts) and are frequently the terminal venues of publications. In addition, discussions between reviewers also takes place in various grant panel reviews (although these occur in face-to-face settings rather than typed forums).*
>
> Like Reviewer 1, in my field this is not standard practice. I would suggest that you include a statement in your Introduction about full papers being reviewed at AI conferences, for the benefit of readers from different fields.

We mention this distinction in our manuscript in the Introduction section as footnote 1. The text is reproduced here:

'Conferences in Computer Science are typically ranked at par or higher than journals, review full-length papers, and are considered to be a terminal publication venue.'

2- Some of your methods are written in the present tense. It may improve the article if you change this and use the past-tense whenever you are describing/explaining your work. Introductory content that describes current thinking etc should stay in the present tense, but I would suggest that when you are describing your study design, survey, analysis etc that you standardize to the past tense.

Thank you for your comment, we have incorporated it in our manuscript and changed the tense where needed.
